# Acylcarnitine profile in Alaskan sled dogs during submaximal multiday exercise points out metabolic flexibility and liver role in energy metabolism

**DOI:** 10.1371/journal.pone.0256009

**Published:** 2021-08-12

**Authors:** Irene Tosi, Tatiana Art, François Boemer, Dominique-Marie Votion, Michael S. Davis

**Affiliations:** 1 Department of Functional Sciences, Fundamental and Applied Research for Animals & Health (FARAH), Faculty of Veterinary Medicine, University of Liège, Liège, Belgium; 2 Biochemical Genetics Laboratory, CHU Sart-Tilman, University of Liège, Liège, Belgium; 3 Equine pole, Fundamental and Applied Research for Animals & Health (FARAH), Faculty of Veterinary Medicine, University of Liège, Liège, Belgium; 4 Department of Physiological Sciences, Oklahoma State University, Stillwater, Oklahoma, United States of America; Medical University of Vienna, AUSTRIA

## Abstract

Alaskan sled dogs develop a particular metabolic strategy during multiday submaximal exercise, allowing them to switch from intra-muscular to extra-muscular energy substrates thus postponing fatigue. Specifically, a progressively increasing stimulus for hepatic glycogenolysis and gluconeogenesis provides glucose for both fueling exercise and replenishing the depleted muscle glycogen. Moreover, recent studies have shown that with continuation of exercise sled dogs increase their insulin-sensitivity and their capacity to transport and oxidize glucose and carbohydrates rather than oxidizing fatty acids. Carnitine and acylcarnitines (AC) play an essential role as metabolic regulators in both fat and glucose metabolism; they serve as biomarkers in different species in both physiologic and pathologic conditions. We assessed the effect of multiday exercise in conditioned sled dogs on plasma short (SC), medium (MC) and long (LC) chain AC by tandem mass spectrometry (MS/MS). Our results show chain-specific modification of AC profiles during the exercise challenge: LCACs maintained a steady increase throughout exercise, some SCACs increased during the last phase of exercise and acetylcarnitine (C2) initially increased before decreasing during the later phase of exercise. We speculated that SCACs kinetics could reflect an increased protein catabolism and C2 pattern could reflect its hepatic uptake for energy-generating purposes to sustain gluconeogenesis. LCACs may be exported by muscle to avoid their accumulation to preserve glucose oxidation and insulin-sensitivity or they could be distributed by liver as energy substrates. These findings, although representing a “snapshot” of blood as a crossing point between different organs, shed further light on sled dogs metabolism that is liver-centric and more carbohydrate-dependent than fat-dependent and during prolonged submaximal exercise.

## Introduction

Alaskan sled dogs are highly aerobic mammals and elite endurance athletes. They are able to sustain a prolonged effort over consecutive days, running at approximately 50% of their maximal oxygen consumption (VO_2max_) [1]. The value of their VO_2max_, even if still inaccurate as difficult to measure, has been estimated at 198 ml·min^-1^·kg^-1^ in moderately trained but unraced yearling sled dogs [2], a value that is among the highest in aerobic mammals [3]. Moreover, as with most canids except for Greyhounds, sled dogs have a predominance of slow-twitch highly oxidative (type I and IIa) muscle fibers [4–6] which store more intramuscular triglycerides (IMTG) and less glycogen than fast fibers. Energy expenditure in sled dogs reaches 12,000 kcal/day [7]; their diet is typically low in carbohydrates (CHO) but high in lipids and proteins. This diet composition could reduce the incidence of musculoskeletal injuries and spare their muscle glycogen (MG) stores, thus postponing fatigue [8, 9].

With these premises, it seems consistent to consider lipids as the main fuel of energy in running sled dogs. This belief has been further supported by scientific research demonstrating a transient but not cumulative MG depletion in sled dogs during multiday exercise, followed by its gradual replenishment despite a limited CHO intake [6, 10]. In parallel, significant IMTG depletion, mainly occurring after the first 140 km of a multiday run, as well as increased post-exercise plasma non-esterified fatty acids (NEFA), ketones and urea, was observed [10, 11]. These findings have strengthened the idea that prolonged submaximal exercise in Alaskan sled dogs would induce metabolic adjustments aiming at attenuating MG use and enhancing the oxidation of non-CHO, extra-muscular substrates. However, substrate shift in Alaskan sled dogs has been shown to be more complex. In fact, recent works have shown an increased capacity to oxidize CHO in parallel to a decreased capacity to oxidize medium-chain fatty acids (MCFA) in sled dogs running a 1,600 km race [12, 13]. Furthermore, conditioned sled dogs show an increased basal and exercise-induced glucose-transport activity [14] as well as a progressive increase in the stimulus for hepatic glucose output during multiday exercise. In fact, hormonal and substrate kinetics, and more specifically an increase in glucagon to insulin ratio, support the idea that in these dogs glucose output would be sustained by gluconeogenesis and possibly by hepatic glycogenolysis [1]. Thus, it has been suggested that increased glucose output would on one side fuel submaximal exercise and on the other be at the origin of the replenishment of MG that has been transiently depleted. These findings have shed new light on exercise metabolism of sled dogs that seems to be CHO-dependent and likely liver-centric during multiday submaximal exercise. Liver function as a source of energy substrates during exercise is still poorly described in humans due to the difficult access to tissue samples. The main role of liver especially during prolonged fasting and exercise is to maintain glucose homeostasis via gluconeogenesis and glycogenolysis. In case of prolonged fasting, prolonged exercise and limited CHO intake, liver uses gluconeogenic substrates as glycerol, lactate and amino acids, generated in the liver itself or delivered to the liver by extrahepatic tissues, to synthetize glucose through gluconeogenesis [15].

L-carnitine is an amino acid derivative that can be obtained by diet and by biosynthesis in mammals [16]. After synthesis, it is released into circulation mainly as free carnitine (C0) and acetylcarnitine (C2, two C atoms). Although physiologically present in all biological fluids, carnitine is most abundant in high energy demanding tissues as liver, skeletal and cardiac muscle [17]. Some tissues such as skeletal muscle cannot synthetize carnitine, so they acquire it from the circulation [16], thus ester patterns found in plasma depend on the uptake and release from peripheral tissues. Carnitine plays an essential role in energy metabolism as it transports long-chain fatty acids (LCFAs) into the mitochondria for ß-oxidation after esterification into long-chain acylcarnitines (LCACs). In fact, LCFAs cannot penetrate mitochondrial membranes whereas short and medium chain fatty acids (SCFAs and MCFAs) cross them by passive diffusion [18]. Once in the mitochondrial matrix, acylcarnitines are reconverted into acyl-CoA and carnitine, but this process is bidirectional, so acylcarnitines can be formed back in the mitochondrial matrix and exported to plasma [19]. Furthermore, carnitine acts as a metabolic regulator by buffering excess acyl-CoA moieties which accumulate in cases of increased fatty acid oxidation (FAO) or of high glycolytic fluxes, becoming deleterious to cellular functions [20, 21]. This “buffer role” preserves a viable pool of free CoA to permit continuation of pyruvate oxidation and a better matching of pyruvate dehydrogenase activity and glycolytic flux [21]. Thus it is clear that carnitine and acylcarnitine function extends to both lipid and CHO metabolism where they maintain metabolic flexibility. Acylcarnitine profile is known in humans to be influenced by metabolic status such as fasting and exercise [22, 23] and by pathologic conditions such as diabetes, obesity, insulin resistance and cardiovascular diseases [24–28].

Acylcarnitine profile determination is currently used in human medicine as a routine screening method for inborn metabolic errors [19, 29]. Research exists describing the effect of exercise on plasma and muscle acylcarnitines during exercise in humans [23, 30–32] and in horses [33–36] undergoing different exercise protocols. Early publications (1980s-1990s) on acylcarnitine kinetics in exercising humans reported that high-intensity exercise, and not low-intensity exercise, was able to alter muscle carnitine and acylcarnitine redistribution [23, 37–41]. On the contrary, plasma changes in carnitine homeostasis were either small, absent or not correlated to muscle changes [23, 31]. However, controversy exists as some investigators identified an increase in circulating MCACs and LCACs in response to exercise [32, 42] and others suggested that circulating acylcarnitines could be the result of an exchange with other organs and tissues such as the hepatic carnitine pool [41, 43]. Recent works assessing acylcarnitine metabolism based on tandem mass spectrometry (MS/MS) and on multiorgan fluxes in different species and in different metabolic conditions (fasting, feeding, exercising) have further underlined how muscle poorly interacts with plasma and other compartments [44–46]. On the other hand, these studies have underlined that other organs/tissues, such as liver and heart, contribute to the circulating levels of acylcarnitines [28, 45–47]. Specifically, liver may distribute acylcarnitines as energy substrates or spill them over from its FAO activity to avoid their accumulation [46]. Thus, it seems consistent to infer from this body of literature that changes in carnitine metabolism are compartmentalized (tissue-specific) and highly dependent on different factors such as exercise workload, metabolic status (feeding, fasting) of the individual and on acylcarnitine chain length. Moreover, plasma acylcarnitine profile would be more dependent on the intervention of other tissues and organs, in particular liver, than on muscle carnitine kinetics, thus further highlighting the role of liver as “metabolic hub” during prolonged exercise. To our knowledge, no publication exists assessing acylcarnitines in endurance dogs. Our aim was to assess the effect of multiday exercise on plasma acylcarnitine profile in conditioned sled dogs in order to contribute to the understanding of their unique exercise metabolism.

## Materials and methods

All procedures were approved by the Oklahoma State University Institutional Animal Care and Use Committee according to the principles outlined in the NIH Guide for the Care and Use of Laboratory Animals. Study design is based on a retrospective analysis of blood samples recovered from 9 conditioned sled dogs recruited for other research protocols [1, 6, 11, 48]. These dogs had completed a 5-day, 800 km run and had been sampled at rest and within 60 min after each 160 km run. Whole biochemistry, as described in another publication [1], had been performed on serum, at rest and after exercise. Main results of biochemistry indicated a lower concentration of insulin and a higher concentration of glucagon after completion of exercise compared to resting samples. Non esterified fatty acids (NEFA), β-hydroxybutyrate (BHB) and glycerol concentrations increased after the first day of exercise in comparison to resting values but they gradually decreased returning to baseline with the progression of exercise. Serum glucose remained stable throughout the trial while lactate decreased significantly.

Heparin plasma samples stored at -80°C were sent to the University of Liège’s Biochemical Genetics Laboratory, Belgium, for acylcarnitine analysis by MS/MS [49]. Practically, plasma proteins were precipitated with a methanol solution containing labelled internal standards. Supernatants were evaporated under nitrogen stream and derivatized with butanolic-HCl. Butylated samples were then reconstituted with water/acetonitrile/formic acid (20/80/0.025) and analyzed with a TQ5500 mass spectrometer (Sciex, Framingham, MA, USA). Acylcarnitine profile analysis included C0, short-chain (SCACs, < 6 carbon atoms), medium chain (MCACs, 6 to 10 carbon atoms), long-chain acylcarnitines (LCACs, > 10 carbon atoms) and hydroxyl- and dicarboxyl-species.

Statistical analysis was performed using a commercial software statistical software (Graphpad Prism 6.0, San Diego, CA, USA). Data were transformed into their natural logarithm (ln) prior to analysis and then an ANOVA test on repeated measures was performed. Given the repeated nature of measures, a Mauchly’s test was run to test the sphericity of data related to each acylcarnitine profile; for acylcarnitine profiles that did not pass the Mauchly’s test of sphericity, a Greenhouse-Geisser correction was applied. A Bonferroni’s post-hoc test was used to realize a multiple comparison between different time points. In all measures, *P*<0.05 was considered significant.

## Results

**Fig 1:**
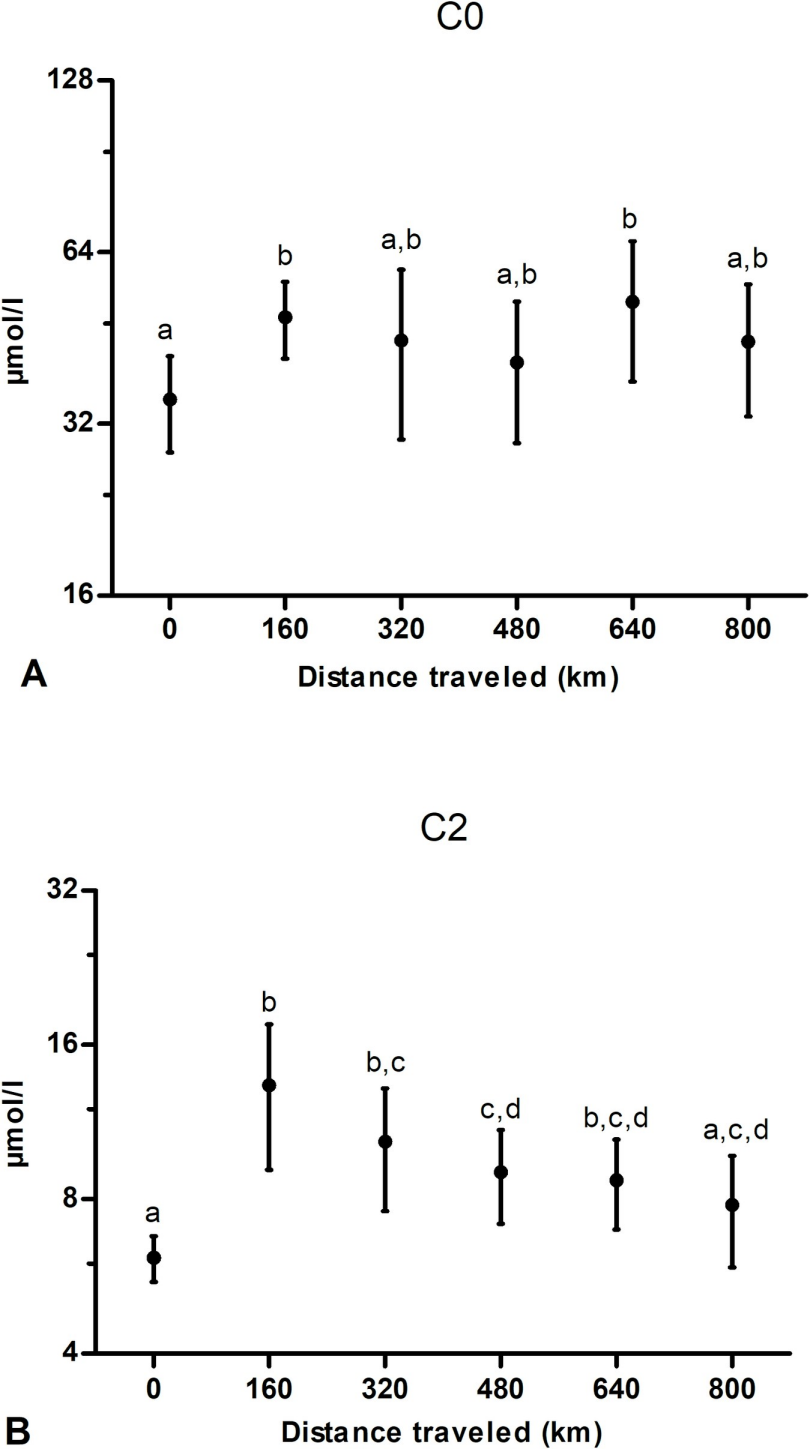
Effect of multiday exercise on plasma free carnitine, or C0 (A) and acetylcarnitine, or C2 (B). Samples were obtained from 9 dogs at 0, 160, 320, 480, 640 and 800 km. Data are displayed as raw data (mean ± SD) on a logarithmic axis. Columns with different superscripts (a, b, c, d) are significantly different between them (*P*<0.05) and not significantly different from columns with the same superscript.

Acetylcarnitine (C2) increased significantly in comparison to baseline (prior to exercise) after the first bout of exercise (160 km), then it decreased progressively over the subsequent bouts of exercise (Fig 1B). Indeed, after 800 km, C2 was not significantly different from pre-exercise value. Free carnitine (C0) was significantly higher than baseline at 160 and 640 km, but not at other time points (Fig 1A).

**Fig 2:**
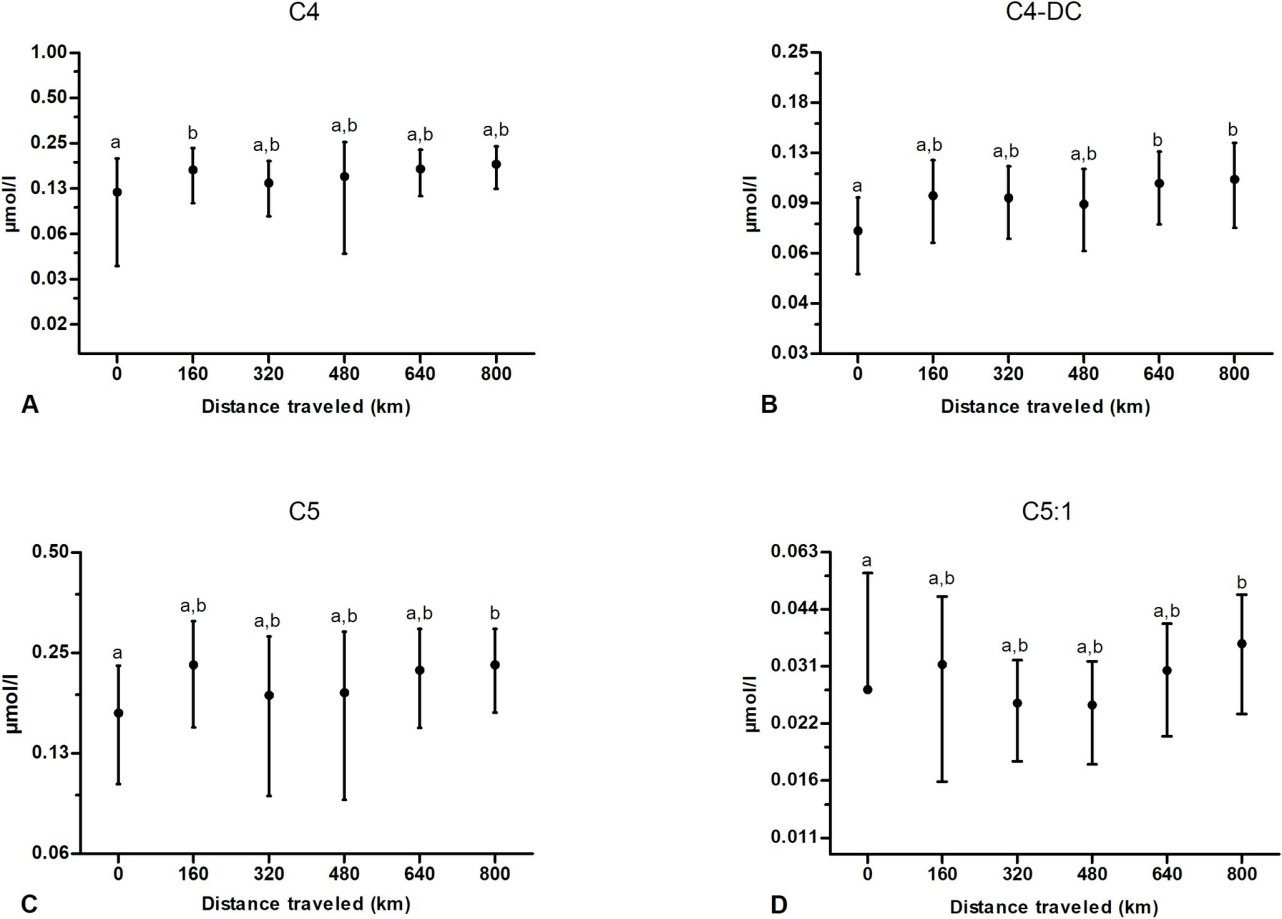
Effect of multiday exercise on some plasma short-chain acylcarnitines (SCACs). Samples were obtained from 9 dogs at 0, 160, 320, 480, 640 and 800 km. Data are displayed as raw data (mean ± SD) on a logarithmic axis. Columns with different superscripts (a, b) are significantly different between them (*P*<0.05) and not significantly different from columns with the same superscript. Missing low error bars correspond to negative values that cannot be displayed on a logarithmic axis.

There was no effect of exercise on C3 and on C3-DC, while C4 was significantly different from baseline after the initial bout of 160 km (Fig 2A). Concerning C4-DC, this profile was significantly higher than prior to exercise after 640 and 800 km (Fig 2B). Similarly, C5 and C5:1 increased significantly compared to baseline after 800 km (Fig 2C and 2D) while there was no significant change for C5-DC and for C5-OH (Table 1).

**Table 1:** Plasma concentrations (μmol/l) of short (SC), medium (MC) and long-chain (LC) acylcarnitines not significantly affected by multiday exercise.

Profile^a^		Rest	160 km	320 km	480 km	640 km	800 km
***SC***	**C3**	0.383±0.194	0.532±0.232	0.455±0.164	0.444±0.271	0.530±0.234	0.525±0.237
	**C3-DC**	0.063±0.052	0.065±0.037	0.050±0.011	0.053±0.010	0.056±0.013	0.069±0.033
	**C5-DC**	0.144±0.091	0.169±0.113	0.129±0.031	0.133±0.056	0.128±0.044	0.158±0.083
	**C5-OH**	0.104±0.047	0.105±0.034	0.083±0.027	0.082±0.013	0.094±0.034	0.100±0.028
***MC***	**C6**	0.043±0.036	0.049±0.026	0.046±0.021	0.049±0.027	0.053±0.030	0.047±0.023
	**C6-DC**	0.053±0.047	0.063±0.036	0.049±0.012	0.047±0.007	0.049±0.010	0.055±0.018
	**C8**	0.053±0.044	0.065±0.036	0.056±0.019	0.047±0.024	0.050±0.018	0.056±0.025
	**C8:1**	0.060±0.052	0.083±0.082	0.067±0.021	0.062±0.020	0.080±0.059	0.084±0.046
	**C8-DC**	0.049±0.034	0.052±0.020	0.057±0.010	0.045±0.007	0.049±0.009	0.046±0.010
	**C10**	0.032±0.024	0.044±0.026	0.039±0.014	0.036±0.018	0.041±0.021	0.042±0.019
***LC***	**C16**	0.412±0.148	0.481±0.165	0.485±0.164	0.471±0.180	0.492±0.147	0.467±0.175

Table legend: ^a^Data are obtained from 9 dogs and reported as mean ± SD. No significant effect of exercise was found at any time point.

**Fig 3:**
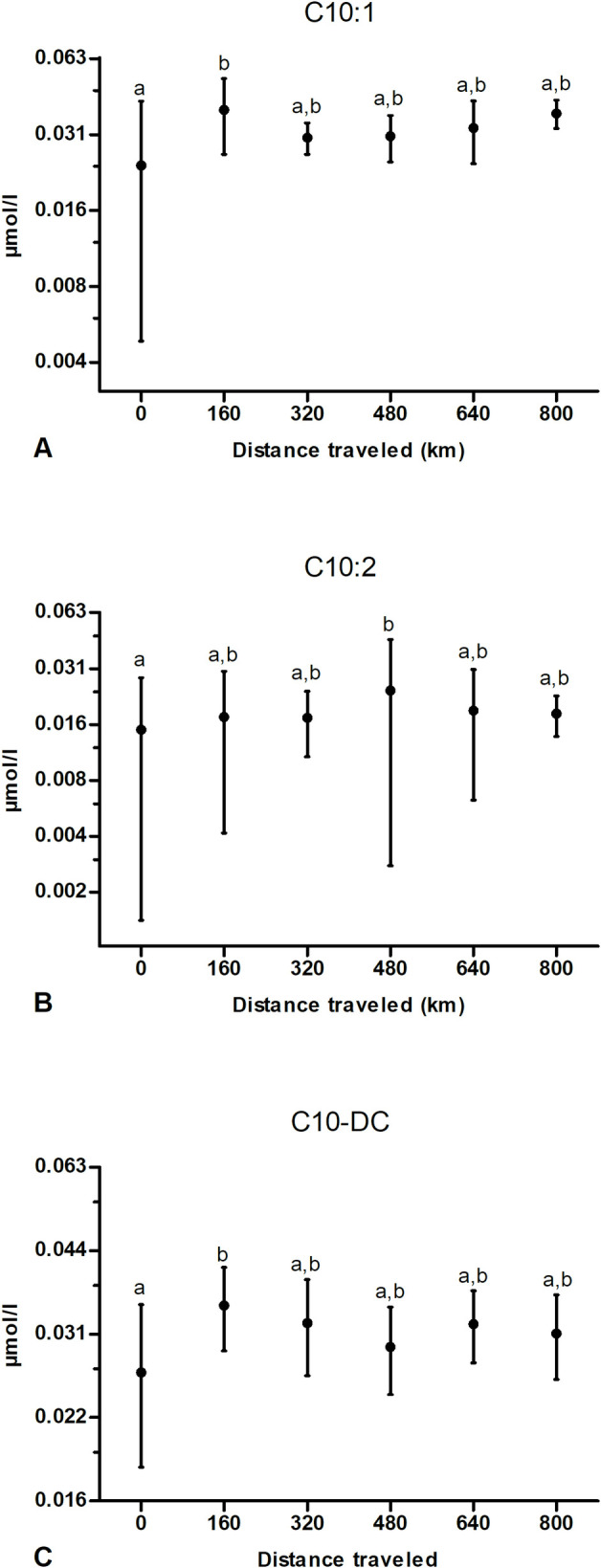
Effect of multiday exercise on some plasma medium-chain acylcarnitines (MCACs). Samples were obtained from 9 dogs at 0, 160, 320, 480, 640 and 800 km. Data are displayed as raw data (mean ± SD) on a logarithmic axis. Columns with different superscripts (a, b) are significantly different between them (*P*<0.05) and not significantly different from columns with the same superscript.

Most MCACs and their hydroxyl- and dicarboxyl-derivatives (C6, C8, C8:1, C6-DC, C8-DC, C10) did not show any significant increase at any time point (Table 1). Some exceptions (Fig 3) were represented by C10:1 and C10-DC (Fig 3A and 3C) that increased significantly after the first 160 km then returning to values not significantly different from baseline. Concerning C10:2, its value was significantly higher than prior to exercise, but only after 480 km (Fig 3B).

**Fig 4:**
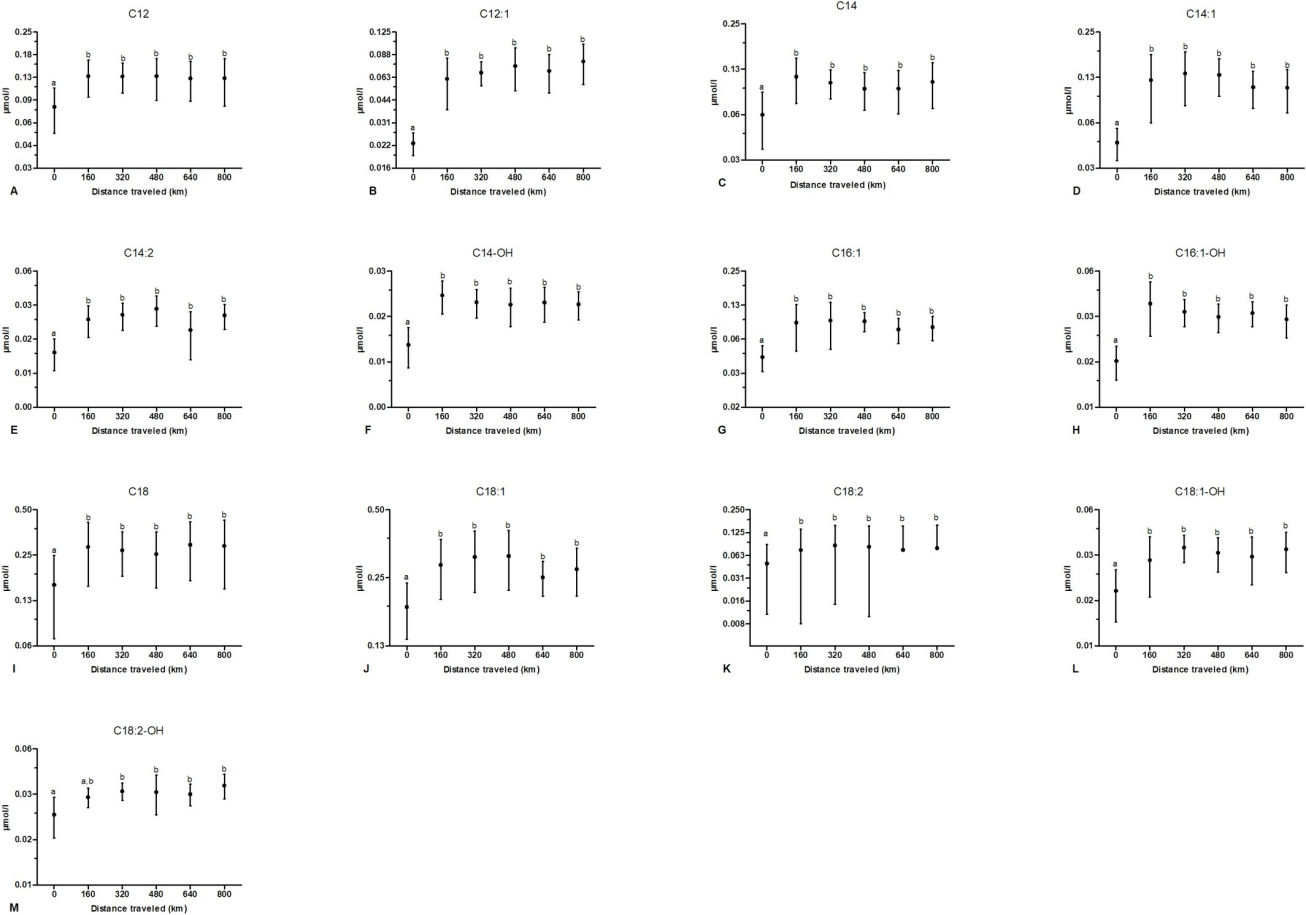
Effect of multiple consecutive days of exercise on plasma long-chain acylcarnitines (LCACs). Samples were obtained from 9 dogs at 0, 160, 320, 480, 640 and 800 km. Data are displayed as raw data (mean ± SD) on a logarithmic axis. Columns with different superscripts (a, b) are significantly different between them (*P*<0.05) and not significantly different from columns with the same superscript. Missing low error bars correspond to negative values that cannot be displayed on a logarithmic axis.

Multiday exercise induced a significant increase in nearly all LCACs (C12, C12:1, C14, C14-OH, C14:1, 14:2, C16:1-OH, C16-OH, C18, C18:1, C18:2, C18:1-OH, C18:2-OH) (Fig 4), except for C16 (Table 1). More precisely, each LCACs profile increased significantly after the first bout of exercise compared to the corresponding pre-exercise values. This increase remained constant throughout the rest of the study, thus being significantly different from pre-exercise values but not different from other time points. It is important to underline that the magnitude of plasmatic increase of acylcarnitine profile with exercise was not homogeneous among different profiles. As an example, variation of SCACs with exercise, even if statistically significant, was less striking (increasing by less than 50% its resting value) compared to the change of C2 that increased of more than 100% of its resting value after 160 km. In the same sense, some LCACs doubled their resting concentration in plasma while other increased (significantly) but to a lesser extent. The biological/energetic impact of these differential variations on the metabolic balance of dogs is unknown.

## Discussion

Contrary to previous hypotheses, there is recent scientific evidence that endurance sled dogs, despite their diet (low-CHO, high-fat and high-protein), the composition of their muscle fibers (predominantly slow and highly oxidative) and the type of exercise they perform (submaximal and prolonged), may actually not be using lipids as their main energy substrate. Indeed, their reliance on CHO as energy source does not decrease but increases with increased fitness [12, 13] in concomitance with an increase in sarcolemmal transport activity of glucose [14]. Moreover, after initial reliance on IMTG and MG during the first day of a multiday exercise challenge, sled dogs experience a metabolic shift away from intramuscular towards blood-borne substrates, and a strong stimulus for glucose output [1]. This metabolic strategy is suggested, as previously described, by an increase of urea production [11], a slight increase in serum glucose and an increase in glucagon/insulin ratio [1].

Our aim was to contribute to the comprehension of this particular substrate management in endurance sled dogs, using plasma acylcarnitines as biomarkers. To our knowledge, this is the first time that acylcarnitines profile has been determined not only in endurance dogs but also in response to a particular metabolic challenge represented by multiday and prolonged exercise.

The most notable result in our study was an increase in almost all LCACs (Fig 4); this increase was significantly different from baseline after the first 160 km and remained steadily constant until completion of the 800 km. Even-chain species from C6 to C22 (MCACs and LCACs) are known to arise from incomplete β-oxidation of fatty acids [50]. Their increase during prolonged exercise and short-term fasting in humans [22, 51] has been related to an increase in NEFA availability provided by lipolysis [52, 53]. Thus their presence in plasma has been suggested to be due to surplus acylcarnitines cleared by muscle to prevent acyl-CoA accumulation in case of increased FAO, to lipids mobilized from lipolysis in form of acylcarnitines or to surplus or newly synthetized acylcarnitines from the liver or other fat oxidizing compartments [45].

Alaskan sled dogs undergoing prolonged submaximal exercise are known from other studies to be in negative caloric balance [1, 7, 10] and to experience active lipolysis as indicated by their glycerol turnover [12]. In another piece of research performed on Alaskan sled dogs exercising over subsequent days, serum NEFA concentration showed, as in our dogs, an initial increase after the first 160 km followed by a progressive decrease despite a progressively increasing glucagon/insulin ratio [1]. Therefore, authors suggested that either a reduction in lipolysis due to body mass reduction (and not insulin-driven) or an increased contraction-mediated extraction of NEFA by skeletal muscle could be at the basis of such NEFA kinetics [1]. However, in our study, LCACs rise observed in plasma was paralleled only after the first 160 km by the rise in circulating NEFA, which afterwards, contrary to LCACs, decreased with continuation of exercise. Thus, it is difficult to relate LCACs rise to an increased availability of NEFA due to lipolysis.

Contracting muscle has often been considered as the main source of plasma acylcarnitines change during exercise because of its increased glucose and lipid metabolism during exercise [23, 30]. Muscle carnitine efflux is likely to be more significant than uptake as muscle has the largest carnitine depot of the body (75% of the total carnitine pool) for FAO, so it necessitates little, even if constant, influx [46]. Skeletal muscle can release acylcarnitines into blood with the aim to remove acyl-CoA moieties that are potentially harmful to cellular functions [54], especially when the provision of energy substrates exceeds the oxidative capacity of the tricarboxylic acid (TCA) cycle. This seems, at a first glance, in contrast with the concept that Alaskan sled dogs are in negative caloric balance [1] and with their switch, due to MG depletion, to extra-muscular non-CHO substrates to sustain energy demand [6, 10]. Nonetheless, it has been demonstrated by recent works that sled dogs exhibit an increased capacity for basal and contraction-mediated sarcolemmal transport of LCFAs and glucose [14] as well as a conditioning-induced clearance of circulating glucose, which result in an enhanced availability of energy substrates in muscle cells. On one hand, these observations underline a discrepancy, or rather a compartmentalized metabolic response, between a systemic negative caloric balance of sled dogs on one side and an “overfed” status of individual muscle cells on the other. On the other hand, they would fit with the idea of muscle “ejecting” surplus acyl-CoA moieties, because of high fluxes of energy substrates massively mobilized during the first bout of 160 km. Moreover, sled dogs have a remarkably high basal insulin sensitivity that is even impressively higher after endurance conditioning [55, 56]. In human medicine, the interrelation between obesity, lipotoxicity and insulin resistance is generally accepted, and the direct or indirect role of acylcarnitines in insulin-resistance has been recently highlighted [24, 50, 57, 58]. Muscle mitochondria are particularly vulnerable to energy overload and serve as the principal lipid sensors in this tissue [50]. Accumulation of intermediates of β-oxidation impair glucose metabolism (by CoA trapping) and mitochondrial performance (by provoking oxidative stress); moreover, it interferes with insulin signaling. Indeed, it has been recently suggested that in human subjects excess LCACs can be converted to metabolites as diacylglycerol and ceramides and activate stress kinases, thus disturbing insulin signaling and contributing to insulin resistance [58, 59]. Looking back at sled dogs, they increase their capacity to transport and to oxidize glucose rather than fatty acids [13, 14], exhibit high values of mitochondrial respiratory capacity [13] and further enhance their insulin-sensitivity [56] during multiday exercise. Therefore, if muscle is the main source of circulating LCACs, we suggest that, even if the initial plasmatic rise in LCACs could still be related to increased FAO rates, their subsequent steady increase would reflect the carnitine-detoxifying role. This function would aim at sustaining CHO metabolism by avoiding CoA trapping, necessary to sustain pyruvate dehydrogenase and thus glucose oxidation, guarantee glucose uptake, conductance to mitochondria and mitochondrial performance.

As previously stated, liver has been shown to play a central role in whole body carnitine metabolism, by distributing it as energy substrate or by spilling it over from its FAO activity [46]. In a recent work [28] assessing the contribution of liver and of skeletal muscle to plasma acylcarnitines in humans, exercise resulted in a systemic increase of LCACs, which were not released (but some even taken up) by skeletal muscle nor by liver. The authors suggested that other oxidizing compartments could be responsible for the elevated circulating levels of LCACs during exercise, and this also could be applied to our findings.

Kidneys can also synthetize carnitine and oxidize acylcarnitines as energy substrate thus regulating the whole body carnitine pool [16]. Nonetheless, a recent study using a porcine transorgan model showed that kidney predominantly clears acylcarnitines up from circulation rather than synthetizing them [46], so its contribution in terms of release may be negligible. Working heart is also known to contribute to changes in MCACs and LCACs in plasma as it uses preferentially fatty acids for ATP production [28, 60].

Exposure to cold is known to trigger in humans and mice systemic changes in lipid metabolism by stimulation of brown adipose tissue metabolic activity, NEFA release by white adipose tissue and hepatic NEFA oxidation [53, 61]. Once released into circulation, NEFA can be directly internalized by brown adipose tissue or indirectly taken up after hepatic esterification in LCACs. In mice, LCACs can be taken up by brown adipocytes as energy fuel for thermogenesis, but they can also improve thermoregulation through the metabolic flux in the liver, producing heat as a byproduct of acylcarnitine synthesis [53]. Sled dogs are exposed to cold temperatures so it could be tempting to hypothesize that LCACs are released constantly into circulation with the aim to favor thermogenesis. Nonetheless, overheating, more than hypothermia, is a common cause of poor performance in sled dogs and it has already been demonstrated that normal working conditions increase dramatically their rectal temperature [62]. Moreover, sled dogs participating in this protocol were spending nearly 50% of the time per day in an exercising state (approximately 10 hours) rather than resting in the cold (approximately 7–8 hours) [11]. Furthermore, blood samples were taken within one hour after the end of exercise, and rectal temperature in running dogs can take more than 30 minutes to drop significantly after exercise [63]. Thus it is unlikely in this context, as in normal racing conditions in which running time largely oversteps resting time, that sled dogs were adopting metabolic strategies to enhance heat production.

In our study, the shorter MCACs as C6, C8, C8:1, C6-DC, C8-DC and C10 (Fig 3) did not show any significant change with exercise. Human literature describes MCACs as the dominating biomarkers of moderate-intensity exercise having the potential biological function to support lipid oxidation during exercise [42]. In a transorgan human model, only MCACs were released by exercising muscles [28] and several of them showed an uptake by the hepato-splanchnic bed. An increase in hydroxyl- and dicarboxyl-acylcarnitines such as C6-DC and C8-DC can reflect an increase in ω-oxidation [64], that is a minor route for FAO taking place in the endoplasmic reticulum of the liver [65]. In humans, omega-oxidation seem to act as a scavenger pathway, to reduce the availability of acyl-CoA metabolites when their intra-cellular level is high [66] due to substrate oversupply. Indeed, these metabolites could be used for the synthesis of potentially lipotoxic species (ceramides and diacyl-glycerols) that could impair insulin signaling [66]. The fact that in our study these profiles did not increase, together with the high insulin sensitivity of these dogs as previously mentioned, may confirm an absence of accumulation of noxious acyl-CoA originating from incomplete β-oxidation. It could be that MCACs in our study were not significantly released by muscle, or that they were promptly picked up from circulation by other organs as energy substrates. This conclusion underlines again the likely predominance of the anabolic function of acylcarnitines in our study, as they represent a pool of C-atoms backbones, available into circulation for the biosynthesis of cellular function and as a potential energy substrate [67]. The catabolic, “detoxifying” function of acylcarnitines allowing efflux of excess acyl groups to alleviate mitochondrial stress [50] seems less likely in our context. A minority of MCACs increased with exercise only at specific time points and depending on the profile. In fact, species as C10:1 and C10-DC increased significantly compared to resting values after the first bout of exercise, then returning to baseline (Fig 3A and 3C), C10:2 increased significantly from baseline only at 480 km (Fig 3B). These punctual significant differences are difficult to explain biologically and need probably a larger number of individuals and further assessment to be confirmed and better understood. In this regard, acylcarnitines classification still lacks consensus and often differs from one publication to another, especially concerning the cutoff between MC and LCACs. The absence of a uniform classification, together with the observation that the kinetics of C12 and C12:1 profiles was more analogous to other LCACs than to MCACs, explains our choice to consider C12 as an LCAC, contrary to other works.

Concerning SCACs (Fig 2), odd-chain carnitine as C3 and C5 derive from amino acids catabolism [50]. In our study, we did not observe any significant change in C3 profile, despite a subjective observation of a tendency to increase, whereas C5 and C5:1 carnitine were significantly higher than baseline but only after 800 km and not at other time points (Fig 2C and 2D); C4-DC followed a similar pattern (Fig 2B). Concerning C4, deriving from both FA and amino acids catabolism, it increased significantly compared to baseline after the first exercise bout (Fig 2A). Previous research performed on the same sled dogs than as those of our study described a decrease in serum protein and an increase in serum urea nitrogen with multiday exercise, which may suggest an enhanced protein catabolism [11]. In that study, serum globulin in particular decreased progressively in a linear fashion with continued exercise. Its concentration was significantly lower than baseline after 480 and 800 km compared with pre-exercise value. Similarly, serum albumin decreased significantly after 320, 480, 640, and 800 km in comparison to its value prior to exercise. Even if our observation remains speculative, the kinetics of SCACs could reflect an increase in protein catabolism as SCACs increase in plasma is simultaneous to the decrease in serum globulins previously observed [11]. It has already been observed that dogs have a high gluconeogenic capacity from precursors as glycerol and lactate (the latter to a small extent) [12, 68, 69], and, probably more importantly, from amino acids [70]. Circulating amino acids derive either from dietary proteins or from endogenous protein catabolism. Commercial diets for sled dogs have a high protein content (>25–30%) [6, 70]; the increase in serum urea nitrogen concentration observed in sled dogs during multiday exercise [11] sustains the idea that an important fraction of these proteins is used for gluconeogenesis [70]. Moreover, a decrease in serum globulin concentration [11], a loss of body mass and an increase in circulating cortisol have been reported in sled dogs during prolonged multiday exercise [1, 11]. Cortisol, among its effects, stimulates proteolysis, thus increasing amino acids availability for gluconeogenesis. Further amino acid availability is induced by glucagon that increases amino acid extraction by the liver [71]. This extraction can be potentially high if proportional to serum glucagon rise observed in exercising sled dogs [70]. In humans, it has been suggested that despite the fact that protein contribution to energy expenditure is minor, exercise induces an increase in amino acids catabolism due to metabolic processes such as hepatic gluconeogenesis and TCA cycle. This phenomenon would partly explain the continuous rise in blood ammonia of humans during prolonged exercise [72]. The recognized depression of protein synthesis in human skeletal muscle during exercise would leave amino acids available for catabolic processes [73]. Recent works demonstrated that Alaskan dogs have a resting mitochondrial protein synthesis rate four times higher than that of resting humans and they maintain this rate during a training program [74]. Nonetheless, this translation of mitochondrial proteins appears to be selective as during exercise training non-mitochondrial (cytosolic and myofibrillar) fractions decrease in Alaskan sled dogs [74]. All these observations, taken together, reflect an increased availability, and subsequent catabolism, of exogenous (dietary) and endogenous (mainly circulating and skeletal-muscle derived) amino acids, as demonstrated by the SCACs profiles of our study, thus matching with the concept of the high gluconeogenic potential and precursor demand of sled dogs.

In our study, C2 (Fig 1B) showed an interesting kinetics, as it increased significantly after the first 160 km in comparison to pre-exercise value, then it decreased progressively with continuation of exercise, returning to a value after 800 km that was not significantly different from baseline. Acetylcarnitine is together with C0 the main circulating form of carnitine released by liver after its synthesis. Cellular enzymes can readily convert carnitine to C2 and back depending on the metabolic needs of the cell, thus these compounds are easily interchangeable [75]. Acetylcarnitine derives from acetyl-CoA, which is the universal product of degradation of different energy substrates converging into their respective catabolic pathway (β-oxidation of fatty acids, catabolism of some amino acids and pyruvate oxidation). Thus C2 can reflect a prolonged and/or massive acetyl-CoA production leading to formation of ketone bodies, thereby becoming a marker of ketosis. In fact, in the mitochondria, when production of acetyl-CoA production oversteps TCA capacity, the acetyl group is transferred to carnitine via the enzyme carnitine acetyltransferase dependent on the equilibrium constant of the enzyme [76]. Therefore, C2 synthesis serves both to maintain a constant pool of free CoA to permit other cellular functions and to buffer excessive and noxious acyl/acetyl groups. Initial rise in C2 observed after 160 km could reflect an acute flow of substrates mobilized from different sources and directed to muscle (muscle glycogen, muscle TG, NEFA released from adipose tissue). Given the similarity between C2, NEFA, BHB and glycerol kinetics and considering the role, among others, of C2 as maker of ketosis, it could be suggested that the decrease of C2 in a linear fashion with continuation of exercise simply mirrors the decreased availability of NEFA and of ketones as a consequence [1]. Nonetheless, C2 carnitine serves also to disseminate energy via acetyl-CoA and represents a 2 C-atoms backbone that can be easily taken up from circulation for energy-generating purposes, especially when, as in this case, stored energy and caloric intake of dogs can no longer meet exercise demand for fuel. Acetyl-CoA in liver activates pyruvate decarboxylase that catalyzes the first step of gluconeogenesis. Submaximal prolonged exercise demands an increase in glucose disposal, so gluconeogenesis has a crucial role in maintaining glucose homeostasis during prolonged exercise as during fasting [77]. A high acetyl-CoA content in hepatic cells together with the rise in serum glucagon of Alaskan sled dogs, as observed by Davis and colleagues [1], both represent powerful stimuli for hepatic gluconeogenesis. Hepatic gluconeogenesis, and possibly glycolysis, would result in sustained glucose output to fuel sustained exercise and to allow MG spare and replenishment [1]. Thus, the constant decrease, after an initial increase, of C2 during multiday exercise can be interpreted as a sign of increasing hepatic uptake of C2 from circulation to stimulate gluconeogenesis rather than as the consequence of a decreased production of ketones.

Free carnitine, C0 (Fig 1A), increased significantly after 160 km and after 640 km in comparison to baseline, but not at other time points. The initial rise in C0 after 160 km seems in accordance with the acute mobilization of energy substrates (IMTG and MG) induced by the first bout of exercise and with the rise in circulating LCACs and C2, thus indicating either an increased rate of (hepatic) synthesis due to increasing demand or to an increased (contracting muscle) release. The following decrease can indicate that C0 release is blunted or that it is being acylated in organs other than contracting muscle (*i*.*e*. liver) as suggested elsewhere [43]. However, it is difficult to explain the significant increase of C0 after 640 km, but it could be related to the observed increased protein catabolism.

We did not compare carnitine/acylcarnitine values of sled dogs to those of non-athletic or sedentary dogs. Plasma carnitine and AC reference values in non-athletic dogs have been reported elsewhere [78–80]. In these works, circulating carnitine has been classified into free and esterified fractions. Interestingly, resting free carnitine concentration, or C0, in the dogs of our study (24–48 μmol/l) seems comparable or slightly higher than reference values obtained from sedentary dogs (12–38 nmol/ml, 9–45 μmol/l) [78–80] and to another population of sled dogs we sampled in an untrained state (12–28 μmol/l)(data not shown). The esterified carnitine fraction generally refers to the sum of C2 and all other short, medium and long-chain profiles; its reference values in non-athletic dogs ranges from 0 to 7 nmol/ml [78, 79] and from 4 to 5 μmol/l in athletic untrained sled dogs (data not show). In our study we presented C2 and other esterified profiles separately. The former ranges from 5 to 7 μmol/l, while all other profiles summed together account only for 1 to 2 μmol/l maximum. Taken into account this calculation, the esterified carnitine fraction in our study seems also quite similar to the corresponding values observed in non-athletic and in untrained dogs. In humans, different metabolic and dietary circumstances, as fasting and long-chain triglycerides load in particular, can affect plasma acylcarnitine levels [51]. Thus, the different composition of diet for sled dogs compared to the diet for sedentary dogs may potentially induce different baseline values but our comparison is descriptive and not sufficiently rigorous to highlight potential differences between the two groups.

Considering the high glucose and CHO-dependence of endurance sled dogs during multiday exercise, as suggested by previous publications and further highlighted by this study, the question could be raised whether if a high-CHO diet would be more beneficial for these dogs than a high-fat diet. A diet rich in fat and proteins, compared to a high CHO-diet, preserves sled dogs from musculo-skeletal injuries and spares glycogen stores [9, 81]. Since dog domestication, humans have imposed a selective pressure that has impacted dog biology, by inducing metabolic and dietary adaptations reflecting co-evolutionary traits of these two species. In both humans and dogs, a copy-number expansion of the pancreatic amylase (*AMY2B*) gene has accompanied the rise and the worldwide spread of agriculture [82], enabling more effective processing of complex carbohydrates. Indeed, pancreatic amylase (AMY2B) catalyzes the breakdown of starch into oligosaccharides, and a high amylase activity is this associated with high copy numbers of the *AMY2B* gene [83]. It has been observed that *AMY2B* copy number distribution in canine breeds follows a pattern matching the geographic spread of prehistoric agriculture. Moreover, few copy numbers have been found in Greenland dogs and Siberian Huskies, populating Arctic regions with no or only recent agricultural practices [84]. Nonetheless, the *AMY2B* copy number can also vary within the same breed, as observed in Siberian Huskies and Alaskan Malamutes [85]. This has been attributed to different hypotheses: to an introgression of alleles from wolves or from other indigenous breeds with low copy number; to a relaxation or to a reinforcement of selection pressure. This relaxation or reinforcement could be the respective results of a switch to a low starch-diet on one side, and of the conservation of a high-starch diet on the other, depending on the local dietary habits of humans after dog breeds migration across the world [85]. Thus, local dietary human habits and/or the introgressive hybridization from wolves may have negatively influenced the ability to process CHO in sled dogs.

## Conclusions

The functional role of plasma acylcarnitines is still unclear. Plasma acylcarnitines provide a snapshot of *in vivo* flux of energy substrates through specific steps of fat, CHO and amino acids catabolism [50], thus the significance of their blood kinetics has to be interpreted with caution. Alaskan sled dogs are impressive fatigue-resistant athletes when submitted to a particular metabolic challenge represented by prolonged multiday exercise in conditions of limited caloric intake. Plasma acylcarnitine profile in sled dogs has shown to be impacted by prolonged multiday exercise in a chain-length dependent manner. Our research represents a piece fitting in a larger puzzle of scientific investigation on exercise metabolism of Alaskan sled dogs. Indeed, our study further highlights the recently underlined key-points of the unique energetic strategy of these dogs, that is 1) extremely metabolically flexible 2) CHO- and glucose-, and not fat-, dependent 3) likely liver-centric. Our study is limited principally by the small number of dogs sampled and by the fact that, due to the retrospective nature of the study, only plasma acylcarnitines could be assessed, and no other analysis on tissues (*i*.*e*. muscle) or fluids (*i*.*e*. urine) could be done. Blood is a “crossing point” where organs (skeletal muscle, liver, heart and kidney) release, take up and exchange acylcarnitines depending on their physiological status, on their respective carnitine turnover rate, and on their response to a given metabolic challenge. Nonetheless, our conclusions remain descriptive and are intended to encourage further investigation specifically of liver role in prolonged submaximal exercise metabolism. Liver seems to cover a role of “metabolic hub” in sustaining prolonged exercise and in maintaining glucose homeostasis and in orchestrating metabolites transit into blood to maintain substrate availability. Even if this is still speculative, an increased hepatic functionality (higher metabolic rates, changes in the hormonal response to exercise or in the hepatic gene expression) may occur in endurance sled dogs as an adaptive response to the metabolic demand dictated by prolonged exercise, and thus privileged and transmitted throughout years of selective breeding.

## Supporting information

S1 File Raw and transformed acylcarnitine serum concentrations obtained from Alaskan sled dogs exercising for 5 days at 160 km/day. (XLS)
